# Isolated Langerhans Cell Histiocytosis of Orbit: A Case Report and Review of the Literature

**DOI:** 10.1155/2018/1529281

**Published:** 2018-04-04

**Authors:** Mayuresh Naik, Anuj Mehta, Neha Mehrotra, Anil Solanki

**Affiliations:** Department of Ophthalmology, VMMC & Safdarjung Hospital, Ring Road, Ansari Nagar, New Delhi 110029, India

## Abstract

A 2-year-old male child presented with a painless progressive mass in the inferolateral aspect of right orbit of three-month duration. Differential leucocyte count revealed raised eosinophil count (13%). On radiological examination, CT scan showed 25 × 27 mm round well-defined smooth-outlined homogenously enhancing extraconal mass arising from the zygomatic bone at the inferotemporal periorbital area of right orbit with bone erosion. Histopathological examination of the incision biopsy revealed characteristic Langerhans cells and immunohistochemical studies were positive for S-100 protein and adenosine deaminase. A diagnosis of Langerhans Cell Histiocytosis (LCH) was made and PET-CT revealed no other foci of uptake anywhere else in the body. The patient received 12 cycles of vinblastine, 0.2 mg/kg body weight, along with oral prednisolone, 1 mg/kg body weight. On completion of three cycles of chemotherapy, a reduction in size of the mass was noticed. A repeat PET scan was done 3 months after completion of chemotherapy did not reveal any activity noted previously.

## 1. Introduction

Langerhans Cell Histiocytosis (LCH) is an uncommon multisystem disorder of unknown etiology, characterized by accumulation of histiocytes in various tissues. It has a variable clinical course, and although it is occasionally seen in adults, it predominantly affects children.

Three clinical forms of LCH have been identified ranging from localized LCH (eosinophilic granuloma), chronic recurring LCH (Hand–Schuller–Christian disease), and acute disseminated LCH (Letterer–Siwe disease).

## 2. Case Report

A 2-year-old male child presented with a painless slowly progressive mass in the inferolateral aspect of right orbit of three-month duration. There was no associated history of fever or any other systemic illness.

General physical examination and systemic examination were normal. Local examination revealed fixed, firm, nontender mass in inferolateral orbit of approximately 25 × 20 mm.

**Figure 1:**
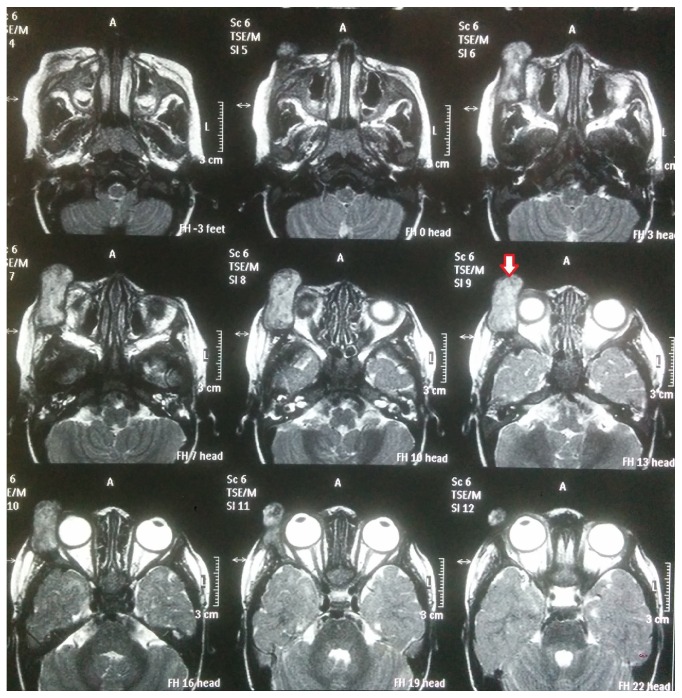
Prechemotherapy MRI scan showing localization and extent of eosinophilic granuloma with bony erosion. Arrow shows the actual pathology, that is, the eosinophilic granuloma.

Differential leucocyte count revealed raised eosinophil count (13%). On radiological examination, CT scan showed 25 × 27 mm round well-defined smooth-outlined homogenously enhancing space-occupying mass arising from the zygomatic bone at the inferotemporal periorbital area of right orbit with bone erosion. The MR imaging showed an exophytic infiltrative mass with irregular margins arising from the anterolateral wall of right orbit. The mass was localized to the extraconal space sparing the intraconal compartment. The lateral rectus muscle was not involved and was distinctly visible and separate from the mass (Figure 1).

**Figure 2:**
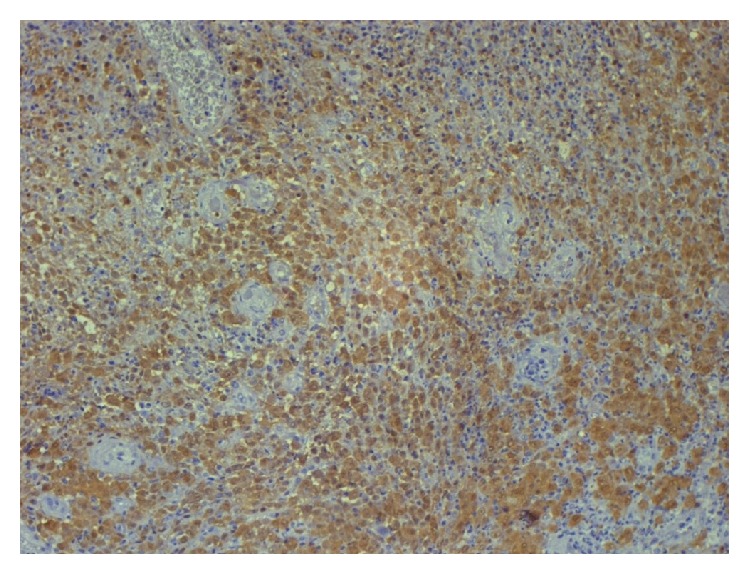
Immunostain specific S-100 positivity on immunohistochemistry.

Incision biopsy of the mass was done and histopathological examination of the specimen revealed characteristic Langerhans cells, 15–20 um in size with discrete nucleolus and homogenous eosinophilic cytoplasm. The immunohistochemical studies were positive for S-100 protein and adenosine deaminase (Figure 2). CD1a positivity was also demonstrated while electron microscopy revealed Birbeck granules.

**Figure 3:**
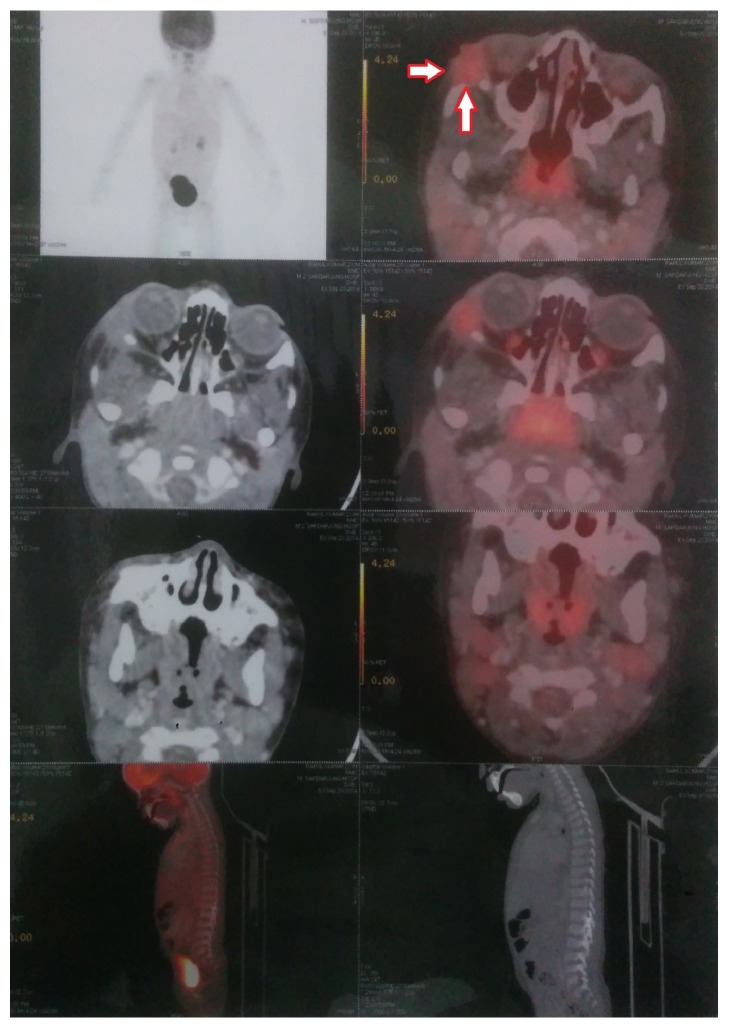
PET-CT scan with arrows marked showing increased uptake suggestive of increased activity at the site of the eosinophilic granuloma (Langerhans Cell Histiocytosis).

A diagnosis of LCH was made and the patient was subjected to PET-CT to identify any other foci. The PET-CT revealed a single focus of uptake in the lateral wall of the orbit (Figure 3). No other foci of uptake were noticed anywhere else in the body.

**Figure 4:**
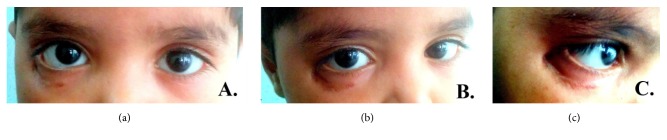
Postchemotherapy clinical photograph of the patient localizing the resolution of the site at inferolateral right orbit.

**Figure 5:**
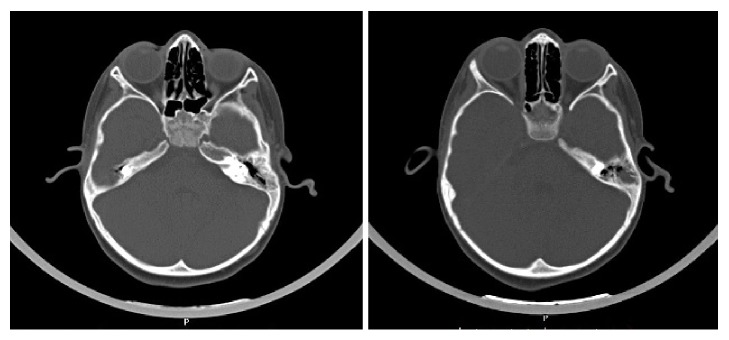
Postchemotherapy CT scans of the patient.

The patient received 12 cycles of vinblastine 0.2 mg/kg body weight along with oral prednisolone 1 mg/kg body weight. On completion of three cycles of chemotherapy, a reduction in size of the mass was noticed (Figures 4 and 5).

A repeat PET scan was done 3 months after completion of chemotherapy. The PET scan did not reveal any activity noted previously.

The patient is currently being followed up for last 2 yrs without any recurrence.

## 3. Discussion

The annual incidence of LCH has been estimated to be 2 to 10 cases per 1 million children aged 15 years or younger [1–3]. The overall incidence of orbital Langerhans Cell Histiocytosis is estimated to be 20%, most commonly as eosinophilic granuloma. Ironically, eosinophilic granuloma is a relatively uncommon entity, accounting for only 1% of all tumor like lesions of bone. Of the total incidence of LCH, 90% have been reported in the head-neck area [1–3]. Furthermore, 25% of these head-neck LCH occur in the orbits. However, involvement of the orbit by Langerhans Cell Histiocytosis accounts for fewer than 1% of all orbital tumors [1–3]. LCH may occur in a spectrum of disease from unifocal unisystem, multifocal unisystem, and multisystem disease and as such, orbital disease may be accompanied by intracranial or systemic involvement. In this context, solitary isolated orbital eosinophilic granuloma is not so common with only a handful of cases reported till date.

Bezdjian et al. reviewed 201 patients from 45 published studies of isolated LCH bony lesions and formulated a systematic algorithm for diagnosis, investigations and management [4]. Mean age at diagnosis of isolated LCH at diagnosis was approximately 8.1 ± 4.3 years while ranging from 2 weeks to 17 years [4–8]. However the youngest reported case of isolated solitary orbital eosinophilic granuloma was a 16-month-old male patient from Iowa [9]. Our patient was 2 years old at presentation and was well within the tenets of the range frames.

LCH occurs predominantly in males (male : female ratio 2 : 1) [4–8]. Patients generally present with swelling (64%), pain and swelling (18%), and just pain (9%) while minor category of patients even presented with torticollis, paresthesia, hearing difficulties, and bleeding. LCH bony lesions are located in the skull (61%), orbit (24%), cervical spine (8%), and mandible (4%) and each of these sites may be a part of either unifocal unisystem, multifocal unisystem, or multisystem disease wherein isolated solitary lesions of the orbit being not so common [4–8]. Our patient presented only with swelling at the inferotemporal periorbital area without evidence of any other foci of eosinophilic granulomas anywhere else in the body.

Treatment modalities available for isolated solitary orbital eosinophilic granuloma include surgical resection, resection with post-op chemotherapy, intralesional steroids followed by resection, radiotherapy, or a combination of two or more modalities with good prognosis while chemotherapy as a first line modality is primarily used only for multisystem disease [10–12]. Bezdjian et al. devised an algorithm for treatment of eosinophilic granulomas reiterating these underlying principles per se [4].

The Histiocyte Society [13, 14] has laid down guidelines for the diagnosis, clinical examination, laboratory, and radiographic evaluation so as to set down the criteria for definitions of organ involvement as well as stratify patient severity into single system or multisystem disease.

They reinstated that the following localizations and disease extent categories are considered indications for systemic therapy [13, 14]:

- SS-LCH (single system LCH) with “CNS-risk” lesions;
- SS-LCH with MFB (multifocal bone lesions);
- SS-LCH with “special site” lesions;
- MS-LCH (multisystem LCH) with/without involvement of risk organs.

The guidelines [13, 14] upheld that treatment duration of 12 months reduces the rate of reactivation as compared to 6 months of total treatment. Patients with MS-LCH at diagnosis can have a variable clinical course. Those without involvement of risk organs, as well as those with involvement of risk organs who respond to standard initial therapy, have an excellent chance of long-term survival. A combination of prednisone (PRED) and vinblastine (VBL) has been proven to be effective treatment with minimal toxicity (6–8) and is therefore the standard initial therapy for all patients in whom systemic therapy is indicated.

Intralesional steroids carry a high rate of recurrence and were therefore avoided [4, 10–12]. Radiation, even though administered to the localized area of involvement, has its accompanying complications, namely, skin necrosis, hair loss, optic nerve damage at such close proximity, neurological damage, and pituitary imbalances [15]; and hence it was decided not to favour radiation as the primary treatment modality. We were then left with the options of using of either localized surgical curettage or systemic chemotherapy. Considering the age of the patient, the localization of the lesion, the size of the granuloma, and the extent of bony erosion, a consultation with the medical oncologist was advised and a trial of systemic chemotherapy to reduce the preoperative size of the granuloma was given. The response to chemotherapy was remarkable with the granuloma literally melting away and being reduced to negligible proportions by the end of the third cycle of chemotherapy. The patient was followed up closely throughout his 12 cycles of vinblastine chemotherapy without evidence of any complications and without evidence of any long-term recurrence.

## 4. Conclusion

Diagnosed as an isolated solitary orbital eosinophilic granuloma, our patient received 12 cycles of vinblastine chemotherapy without evidence of any complications and without evidence of any recurrence over the 2 years of follow-up. In our opinion, chemotherapy is a relatively safe and effective treatment option in paediatric patients and may be considered as the primary modality of choice in isolated solitary as well multisystem LCH.

## Additional Points

*Key Messages*. (1) What is already known about this subject? Treatment modalities available for isolated solitary orbital eosinophilic granuloma include surgical resection, resection with post-op chemotherapy, intralesional steroids followed by resection, radiotherapy, or a combination of two or more modalities. (2) What are the new findings? Involvement of the orbit by Langerhans Cell Histiocytosis accounts for less than 1% of all orbital tumors. In this context, solitary isolated orbital eosinophilic granuloma is not so common with only a handful of cases reported till date. Systemic chemotherapy is a relatively safe and effective treatment option in paediatric patients and may be considered as the primary modality of choice in isolated solitary as well multisystem LCH. (3) How might these results change the focus of research or clinical practice? This case report truly represents a paradigm shift in the management of solitary unifocal isolated LCH from invasive disfiguring surgical curettage to cheap, safe, simple, and effective chemotherapy.
